# Texture Analysis Based on Sagittal Fat-Suppression and Transverse T2-Weighted Magnetic Resonance Imaging for Determining Local Invasion of Rectal Cancer

**DOI:** 10.3389/fonc.2020.01476

**Published:** 2020-08-18

**Authors:** H. C. Lu, F. Wang, J. D. Yin

**Affiliations:** ^1^School of Medicine and Bioinformatics Engineering, Northeastern University, Shenyang, China; ^2^Department of Radiology, Shengjing Hospital of China Medical University, Shenyang, China

**Keywords:** rectal cancer, local invasion, imaging informatics, intelligence, texture analysis

## Abstract

**Background:** Accurate evaluation of local invasion (T-stage) of rectal cancer is essential for treatment planning. A search of PubMed database indicated that the correlation between texture features from T2-weighted magnetic resonance imaging (T2WI) (MRI) and T-stage has not been explored extensively.

**Purpose:** To evaluate the performance of texture analysis using sagittal fat-suppression combined with transverse T2WI for determining T-stage of rectal cancer.

**Methods:** One hundred and seventy-four rectal cancer cases who underwent preoperative MRI were retrospectively selected and divided into high (T3/4) and low (T1/2) T-stage groups. Texture features were, respectively, extracted from sagittal fat-suppression and transverse T2WI images. Univariate and multivariate analyses were conducted to determine T-stage. Discrimination performance was assessed by receiver operating characteristic (ROC) analysis.

**Results:** For univariate analysis, the best performance in differentiating T1/2 from T3/4 tumors was achieved from transverse T2WI, and the area under the ROC curve (AUC) was 0.740. For multivariate analysis, the logical regression model incorporating the independent predictors achieved an AUC of 0.789.

**Conclusions:** Texture features from sagittal fat-suppression combined with transverse T2WI presented moderate association with T-stage of rectal cancer. These findings may be valuable in selecting optimum treatment strategy.

## Introduction

Colorectal cancer is the third leading cause of cancer worldwide, and rectal cancer accounts for 30–35% of colorectal cancer cases. Accurate assessment of rectal cancer features is essential for determining the optimal treatment strategy to reduce the risk of local recurrence and improve patient survival (1, 2). The choice of treatment depends on tumor stage, and rectal tumors are staged according to pathological features, including the extent of tumor invasion (pathological T-stage) (3, 4). Due to the noninvasive advantage in assessing tumor microcirculation, high-resolution magnetic resonance imaging (MRI) is widely applied to stage primary rectal cancer before treatment (5). However, the ability of MRI in discriminating stage T2 from stage T3 tumors is limited because tumor penetration through the rectal muscular wall is similar to the peritumoral inflammatory reaction (6). Tissue edema, fibrosis, and inflammation may decrease the accuracy of MRI after neoadjuvant chemoradiotherapy (NACT) (7).

An emerging quantitative method for imaging informatics, texture analysis (TA), is used to quantitatively describe the spatial distribution of gray values within images, and it can detect image patterns that are unrecognizable or indistinguishable to the human eyes (8). TA extracts high-throughput information to quantify tumor heterogeneity within a defined region of interest (ROI) (9). The most commonly used texture features can be stratified according to the statistical order of the voxel information encoded within the image, including first-order (also known as histogram features), second-order [run-length matrix (GRLM) and gray-level co-occurrence matrix (GLCM) features], and higher-order (structural and transformation-based features) texture features (8). Certain texture features extracted from MRI or computed tomography can be used for tumor diagnosis, preoperative risk stratification, and prediction of survival (10–12). Studies of rectal cancer suggest that texture features are useful for predicting pathological complete response after NACT (13–15).

Accurate evaluation of rectal adenocarcinoma before therapy is essential because treatment strategies need to be personalized following the histopathological results. Texture parameters of primary tumors on T2-weighted MRI (T2WI) are associated with lymph node metastasis (N stage) (16). Lu et al. (4) reported that texture features extracted from apparent diffusion coefficient (ADC) maps may be helpful in predicting rectal cancer T-stage. However, a search of the PubMed database indicated that the correlation between T2WI texture features and the extent of local invasion has not been investigated extensively in rectal cancer. There are no studies reporting the use of texture features derived from sagittal fat-suppression combined with transverse T2WI in T-stage determination of intelligence, which is a common sequence in the evaluation of rectal cancer (17).

## Materials and Methods

### Ethics

Our study was performed in accordance with the ethical standards of the World Medical Association Declaration of Helsinki, and approved by Ethics Committee of Shengjing Hospital of China Medical University (Project Identification Code: 2020PS011K) (date of approval: 7 January 2020).

### Patient Cohorts

**Figure 1:**
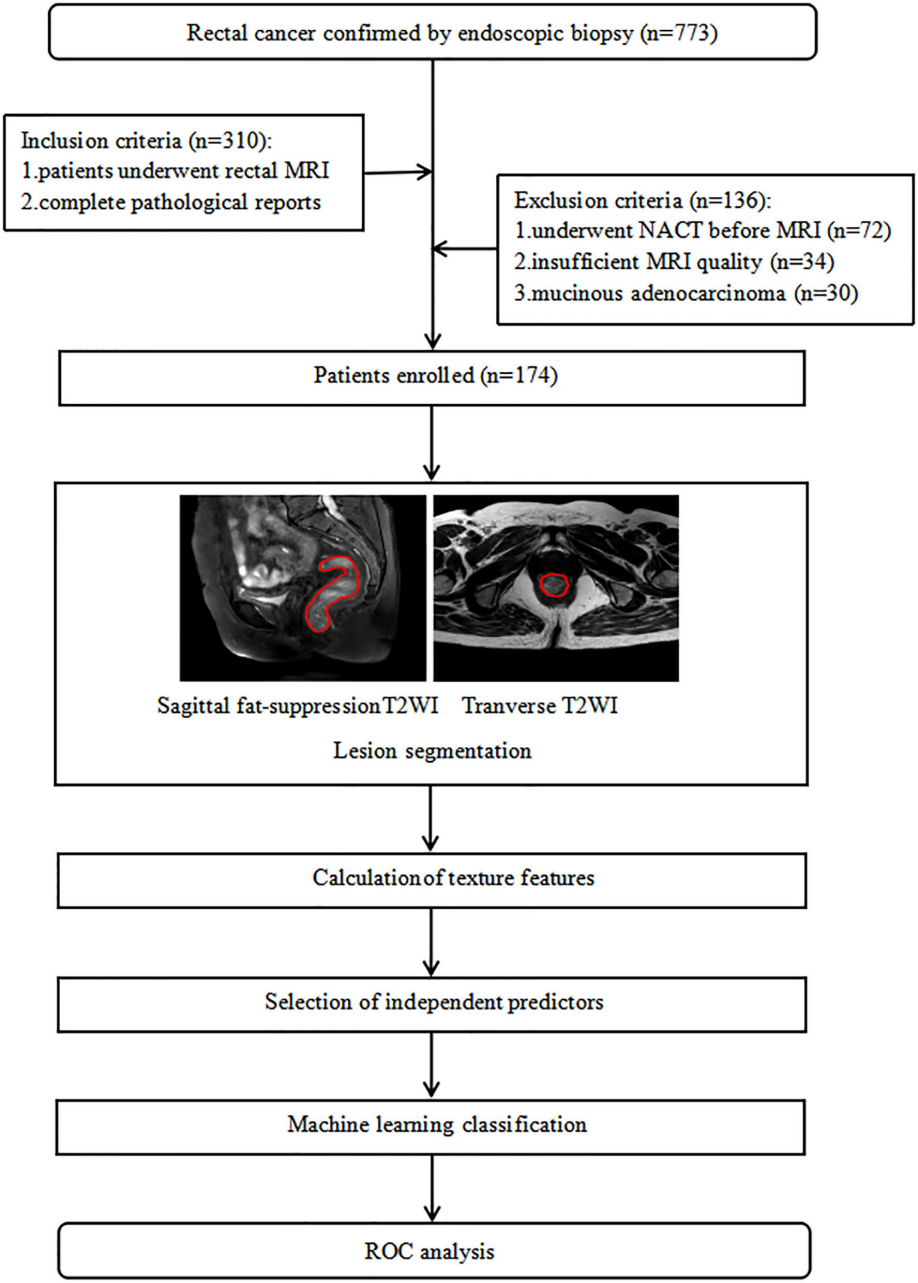
Flowchart of the method used for T-stage classification.

We reviewed 773 consecutive patients with rectal adenocarcinoma confirmed by endoscopic biopsy or postoperative pathological examination between January 2018 and November 2019 in our hospital. Patients who underwent rectal MRI were enrolled (*n* = 310). One hundred and thirty-six patients were excluded because of (1) NACT before MRI examination; (2) poor image quality caused by apparent motion artifacts; or (3) pathologically proven mucinous adenocarcinoma. Finally, 174 eligible patients were included in the study. Clinical data including sex, age, maximum tumor diameter, tumor location, degree of tumor differentiation, and N stage were collected. Patients were divided into high (T3/4) and low (T1/2) T-stage groups according to the pathological results. The flowchart of this study is shown in Figure 1.

### MR Image Acquisition

All MRI examinations were conducted using a 3.0 T machine (Ingenia 3.0, Philips Medical System, Best, The Netherlands). A surface coil of eight-channel phased-array was applied to patients in the supine position during imaging. Both bowel preparation and intravenous antispasmodic agents were not executed. High-resolution rectal MRI protocols included sagittal fat-suppression and transverse T2WI as well as diffusion-weighted imaging (DWI). Acquisition parameters for transverse T2WI were showed below: repetition time (TR)/echo time (TE), 2200/65 ms; flip angle, 90°; matrix size, 288 × 288; field of view (FOV), 250 × 250 mm^2^; slices, 20; slice thickness, 5 mm; spacing between slices, 0.5 mm; NSA, 2. Parameters for sagittal fat-suppression T2WI were: TR/TE, 2200/90 ms; flip angle, 90°; fat suppression, SPAIR; matrix size, 300 × 300; FOV, 250 × 250 mm^2^; slices, 40; slice thickness, 3 mm; spacing between slices, 0.3 mm; NSA, 3.

### Image Segmentation

Two radiologists who had 10 years of experience in interpreting pelvic MRI conducted the lesion segmentation independently, who were blinded to the pathological results during the image reading. They imported the images into a processing software (ImageJ; National Institutes of Health, Bethesda, MD, USA), and determined lesions as local mass or abnormal wall thickening that presented intermediate signal intensity on T2WI, hypointensity on the ADC map, and hyperintensity on DWI. ROIs were manually drawn along the border of the lesion on the sagittal fat-suppression and transverse T2WI slice showing the maximum lesion diameter with reference to DWI and ADC maps. Apparent regions of necrosis, luminal contents, or gas were avoided to minimize bias.

### Texture Analysis

TA was performed on ROIs from sagittal fat-suppression and transverse T2W images using in-house software programmed with MATLAB 2018a (Mathworks, Natick, MA, USA). Twelve texture features were calculated from each type of image including: (1) histogram parameters; (2) GLCM parameters; (3) GRLM parameters; and (4) discrete wavelet transformation (DWT) parameters. A total of 24 features were derived for each case. Table 1 provides the specific information about those texture features.

**Table 1:** Detailed information on texture features used in this study.

**Features**	**Descriptions**
**Histogram parameters**	
Skewness (SKE)	Asymmetry of intensity level distribution
Kurtosis (KUR)	Peakedness of intensity level distribution
**GLCM parameters^a^**	
Correlation (CORR)	Image complexity
Dissimilarity (DISS)	Local contrasts
Entropy (ENTR)	Randomness of the intensity level distribution
**GRLM parameters^b^**	
Long run emphasis (LRE)	Distribution of long runs
Gray level non-uniformity (GLN)	Similarity of the gray level values
Low gray-level run emphasis	Distribution of low gray level values
(LGLRE)	
**DWT parameters^c^**	
Harr-L	Low frequency components of Harr transform
Harr-H	Horizontal components of Harr transform
Harr-V	Vertical components of Harr transform
Harr-D	Diagonal components of Harr transform

a *GLCM, gray-level co-occurrence matrix. Each GLCM parameter was calculated with a distance of 1 and four angles (0°, 45°, 90°, and 135°), and the average was used as the feature value*.

b *GRLM, gray-level run-length matrix. Each GRLM parameter was calculated with four angles (0°, 45°, 90°, and 135°), and the average was used as the feature value*.

c *DWT, discrete wavelet transformation. DWT parameter was calculated with two layers and three directions (horizontal, vertical, diagonal) to produce low and high frequency components, and second layer components were extracted for texture analysis*.

### Statistical Analysis

Categorical variables (sex, N stage, degree of tumor differentiation, and tumor location) were compared between T1/2 and T3/4 groups utilizing the chi-square or *Fisher's* exact test. Quantitative data (maximum tumor diameter, age, and texture parameters) were first tested by Kolmogorov-Smirnov test to determine if samples presented a normal distribution. If the distribution was not normal (*P* < 0.05), Mann-Whitney *U* test was used to compare parameters between T1/2 and T3/4 stages. Otherwise, independent-sample *t*-test was used. Independent factors predicting T3/4 tumors after collinearity diagnosis were analyzed by multivariate logistic regression. A variance inflation factor (VIF) > 10 indicated the existence of collinearity. In order to evaluate the correlation between features and T-stages, Spearman correlation analysis was executed.

Univariate and multivariate analyses were performed to determine T-stage. Receiver operating characteristic (ROC) theory was applied to assess the discrimination performance by measuring the area under the ROC curve (AUC), which was achieved by a professional statistics software MedCalc (version 14.10.20, http://www.medcalc.org/). The optimal threshold was determined by the maximum Youden index, and the specificity and sensitivity were automatically provided. The statistical significance of differences among AUCs was investigated using Delong method (18).

In this study, we also investigated the intraobserver variability of features extracted by two radiologists using intraclass correlation coefficients (ICCs, 0.81–1, excellent agreement; 0.61–0.8, good agreement; 0.41–0.6, moderate agreement; and 0–0.4, poor agreement).

All statistical analyses were conducted using SPSS 22.0 (IBM Corporation, Armonk, NY, USA), and *P* < 0.05 was regard as a statistically significant difference.

## Results

### Patient Characteristics

**Figure 2:**
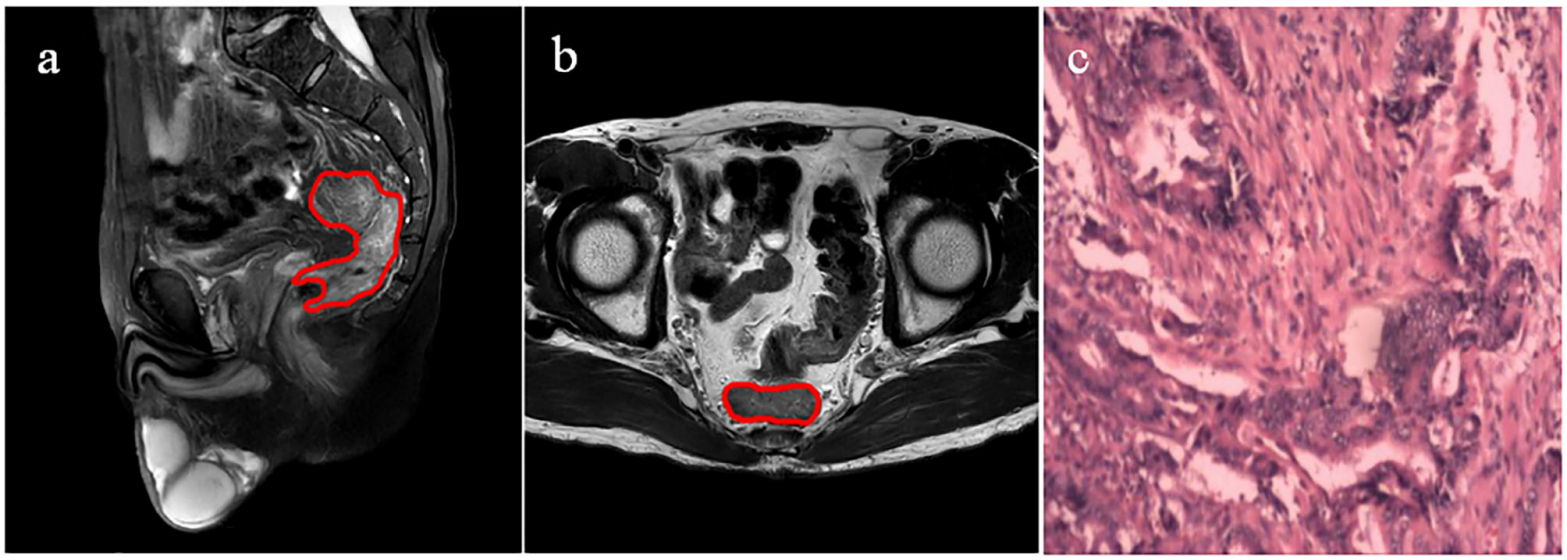
ROI segmentation in a randomly selected case based on sagittal fat-suppression and transverse T2WI. **(a)** Lesion ROI delineated on the sagittal fat-suppression T2WI. **(b)** Lesion ROI delineated on the routine transverse T2WI. **(c)** Pathological result.

The clinical and pathological characteristics of T1/2 and T3/4 cases are listed in Table 2. There were no significant differences between the two groups in sex (*P* = 0.567), age (*P* = 0.537), tumor location (*P* = 0.078), maximum tumor diameter (*P* = 0.673), degree of tumor differentiation (*P* = 0.210), and N stage (*P* = 0.632). A case randomly selected was used to illustrate the segmentation of lesion ROI (Figure 2).

**Table 2:** Clinical and pathological characteristics of patients for identifying the T-stage of rectal cancer.

**Characteristics**	**T-stage**		***P*-value**
	**T1/2**	**T3/4**	
**Total patients**	62 (35.6)	112 (64.4)	/
**Gender**			0.567^a^
Male	36 (58.1)	70 (62.5)	
Female	26 (41.9)	42 (37.5)	
**Mean age (years)^b^**	64.9 (57-83)	65.2 (54-92)	0.537^c^
**Tumor location**			0.078^a^
Upper rectum	24 (38.7)	26 (23.2)	
Middle rectum	26 (41.9)	64 (57.1)	
Lower rectum	12 (19.4)	22 (19.7)	
**Maximum diameter of tumor (cm)**	3.9	4.1	0.673^d^
**Tumor differentiation**			0.210^e^
Moderate to high	58 (93.5)	98 (87.5)	
Low	4 (6.5)	14 (12.5)	
**N stage**			0.632^a^
N0	35 (56.4)	59 (52.7)	
N1/2	27 (43.6)	53 (47.3)	

a *Variables were tested using the χ^2^ test*.

b *Mean value (range)*.

c *Variables were tested using independent sample t-test*.

d *Variables were tested using Mann-Whitney U test*.

e *Variables were tested using Fisher's exact test*.

*Unless otherwise indicated, variable are expressed as frequencies (percentage)*.

### Univariate Analysis

The statistical results of texture features extracted from T1/2 and T3/4 tumors are shown in Table 3. The texture parameters measured from the two sets of ROIs independently delineated by two radiologists using sagittal fat-suppression and transverse T2W images showed excellent agreement (ICCs, 0.832–0.927). DISS, ENTR, GLN, and LGLRE extracted from sagittal fat-suppression and transverse T2WI were significantly higher for T3/4 than for T1/2 tumors. T3/4 tumors had significantly lower Harr-V extracted from sagittal fat-suppression T2WI than T1/2 tumors.

**Table 3:** Comparison of extracted texture features for T1/2 and T3/4 tumors.

**Image**	**Features**	**T-stage**		***P*-value**
		**T1/2 (*n* = 31)**	**T3/4 (*n* = 56)**	
**Sagittal fat-suppression T2WI**	SKE	0.023 ± 0.398	0.066 ± 0.459	0.435^a^
	KUR	3.260 ± 0.644	3.211 ± 0.725	0.677^b^
	CORR	0.956 ± 0.016	0.957 ± 0.015	0.598^b^
	DISS	0.035 ± 0.017	0.049 ± 0.019	0.001^a^
	ENTR	0.151 ± 0.084	0.209 ± 0.081	0.002^a^
	LRE	2.443 ± 0.521	2.542 ± 0.586	0.167^b^
	GLN	6.491 ± 6.126	11.564 ± 8.952	0.017^a^
	LGLRE	0.537 ± 0.113	0.579 ± 0.082	0.019^a^
	Harr-L	9.254 ± 0.259	9.308 ± 0.231	0.321^b^
	Harr-H	0.168 ± 0.095	0.173 ± 1.063	0.439^a^
	Harr-V	0.219 ± 0.082	0.181 ± 0.071	0.014^a^
	Harr-D	0.039 ± 0.027	0.037 ± 0.025	0.579^a^
**Transverse T2WI**	SKE	0.455 ± 0.435	0.624 ± 0.653	0.113^b^
	KUR	4.166 ± 1.316	4.926 ± 3.386	0.362^a^
	CORR	0.933 ± 0.021	0.935 ± 0.028	0.529^a^
	DISS	0.031 ± 0.008	0.041 ± 0.014	0.002^b^
	ENTR	0.109 ± 0.034	0.156 ± 0.069	0.001^b^
	LRE	1.665 ± 0.435	1.595 ± 0.515	0.298^b^
	GLN	5.018 ± 2.942	7.071 ± 5.356	0.002^a^
	LGLRE	0.545 ± 0.056	0.583 ± 0.058	0.026^b^
	Harr-L	8.973 ± 0.258	8.969 ± 0.347	0.976^b^
	Harr-H	0.263 ± 0.092	0.256 ± 0.123	0.651^b^
	Harr-V	0.236 ± 0.077	0.249 ± 0.104	0.562^b^
	Harr-D	0.065 ± 0.032	0.062 ± 0.029	0.551^a^

a *Mann-Whitney U test, data represent the median ± interquartile range*.

b *Independent sample t-test, data represent the mean ± SD*.

**Figure 3:**
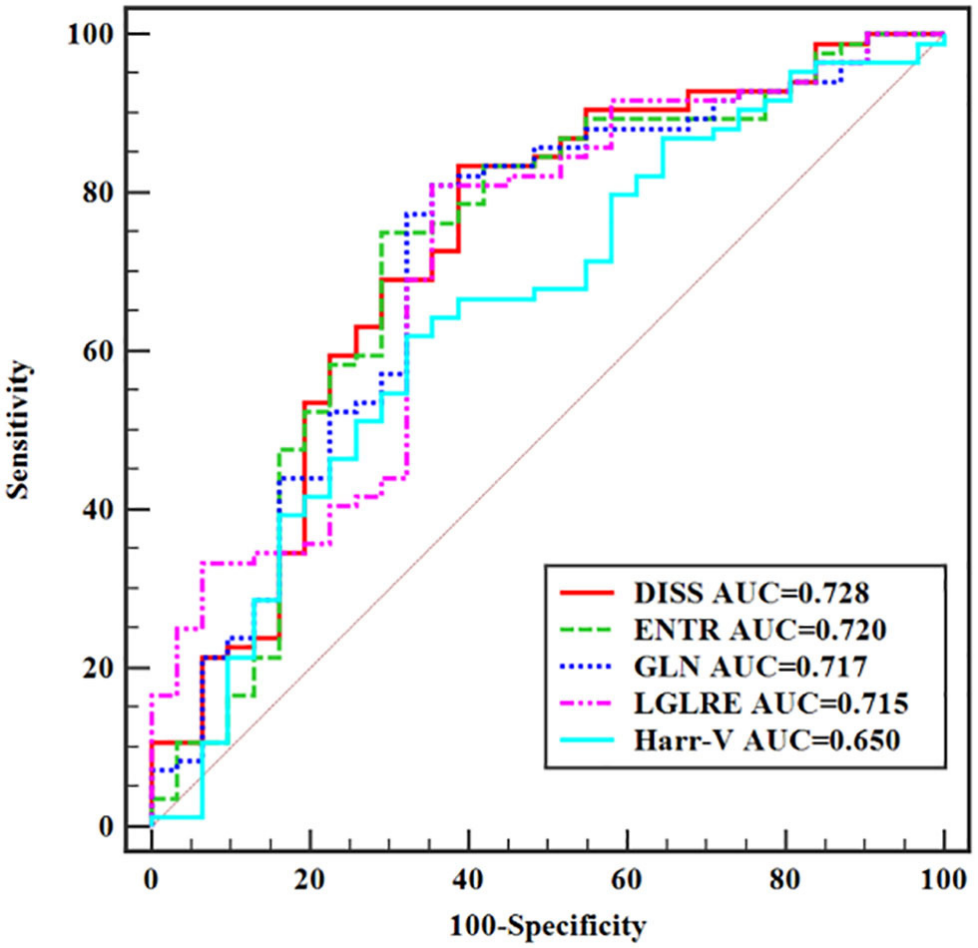
ROC curves of statistically significant texture features extracted from sagittal fat-suppression T2WI for predicting T-stage.

The diagnostic performance of each significantly different feature is shown in Table 4. At cutoff values of 0.029 for DISS, 0.149 for ENTR, 7.202 for GLN, 0.549 for LGLRE, and 0.187 for Harr-V, sagittal fat-suppression T2WI achieved an AUC of 0.728 [95% confidence interval (CI), 0.637–0.807], 0.720 (95% CI, 0.629–0.800), 0.717 (95% CI, 0.625–0.797), 0.715 (95% CI, 0.623–0.795), and 0.650 (95% CI, 0.566–0.737), respectively. Sensitivities and specificities were 83.33 and 61.29% for DISS, 75.0 and 70.97% for ENTR, 80.95 and 64.52% for GLN, 80.95 and 64.52% for LGLRE, and 61.90 and 67.74% for Harr-V. In the pairwise comparison of AUCs, all *P* values were > 0.05. The corresponding ROC curves are shown in Figure 3.

**Table 4:** Diagnostic performance of significantly different texture features for differentiating T1/2 from T3/4 stage rectal cancer.

**Image**	**Features**	**AUC**	**Sensitivity (%)**	**Specificity (%)**	**95% CI**	**Cutoff value**
**Sagittal fat-suppression T2WI**	DISS	0.728	83.33	61.29	0.637–0.807	0.029
	ENTR	0.720	75.00	70.97	0.629–0.800	0.149
	GLN	0.717	80.95	64.52	0.625–0.797	7.202
	LGLRE	0.715	80.95	64.52	0.623–0.795	0.549
	Harr-V	0.650	61.90	67.74	0.566–0.737	0.187
**Transverse T2WI**	DISS	0.730	73.81	64.52	0.640–0.809	0.031
	ENTR	0.740	71.43	70.97	0.650–0.818	0.116
	GLN	0.720	67.86	67.74	0.629–0.800	6.121
	LGLRE	0.696	77.38	58.06	0.604–0.779	0.546

**Figure 4:**
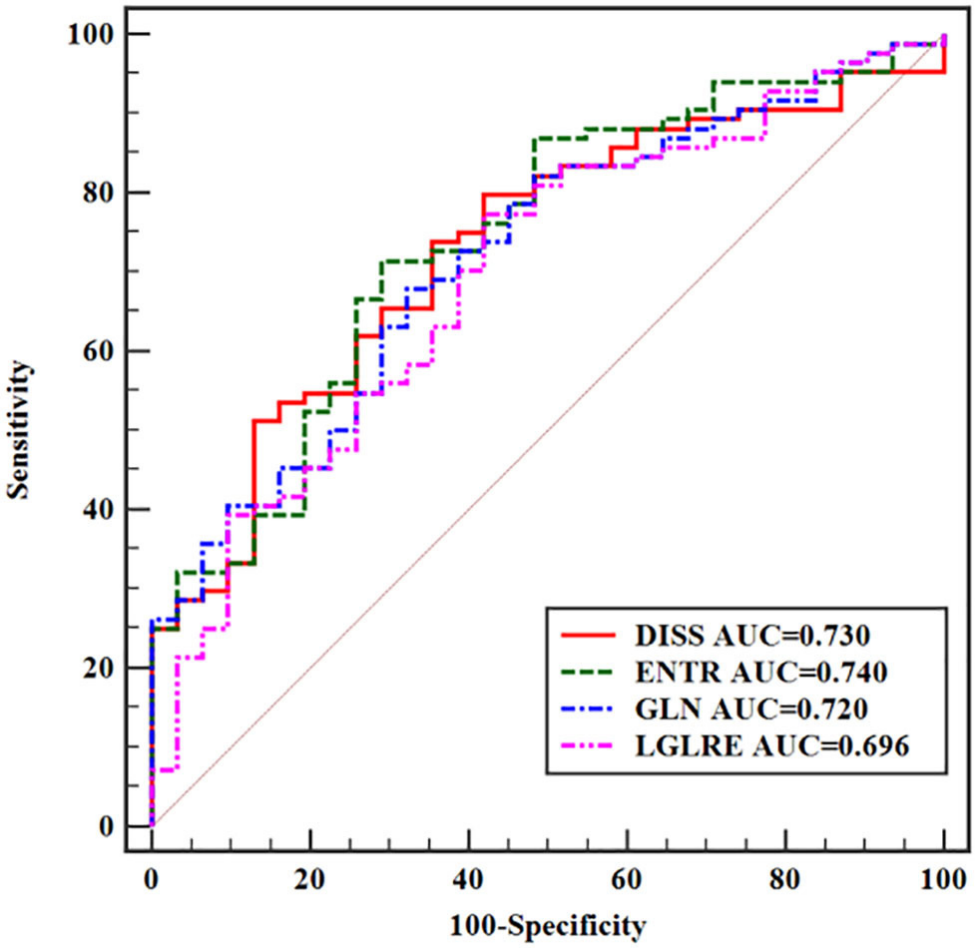
ROC curves of statistically significant texture features extracted from transverse T2WI for predicting T-stage.

With cutoff values of 0.031 for DISS, 0.116 for ENTR, 6.121 for GLN, and 0.546 for LGLRE, transverse T2WI achieved AUCs of 0.730 (95% CI, 0.640–0.809), 0.740 (95% CI, 0.650–0.818), 0.720 (95% CI, 0.629–0.800), and 0.696 (95% CI, 0.604–0.779), respectively. Sensitivities and specificities were 73.81 and 64.52% for DISS, 71.43 and 70.97% for ENTR, 67.86 and 67.74% for GLN, and 77.38 and 58.06% for LGLRS, respectively. There was still no significant difference between each two discrimination performances. The corresponding ROC curves are shown in Figure 4.

### Multivariate Analysis

**Figure 5:**
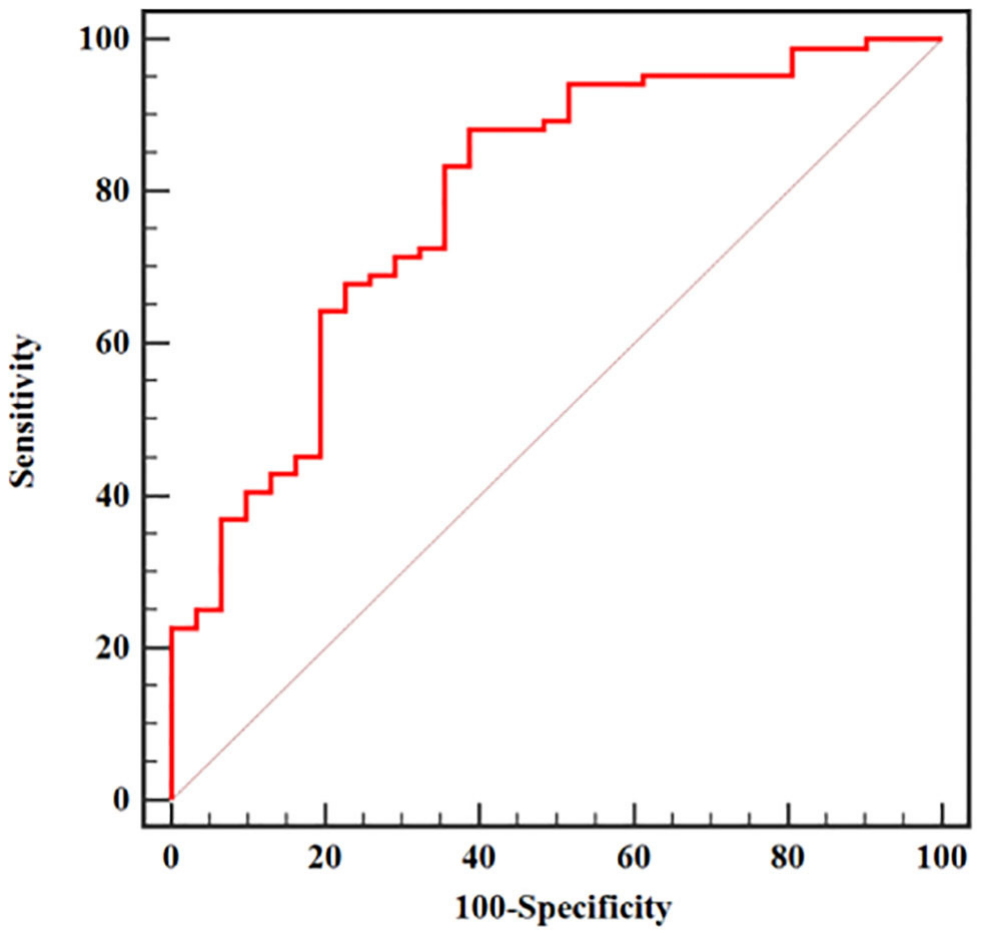
ROC curve of a model for predicting T-stage based on multivariate logistic regression analysis.

The results of multivariate analysis are shown in Table 5. Because there was a strong collinearity (VIF = 18.555) between GLN from sagittal fat-suppression T2WI and other features, GLN was excluded to reduce the effect of collinearity. The cutoff values from the ROC curve analysis were used to convert the texture features into categorical variables for inclusion in the logistic model. Eight variables were applied to the final logistic model: DISS (< 0.029 or > 0.029), ENTR (< 0.149 or > 0.149), LGLRE (< 0.549 or > 0.549), Harr-V (< 0.187 or > 0.187) from sagittal fat-suppression T2WI, and DISS (< 0.031 or > 0.031), ENTR (< 0.116 or > 0.116), GLN (< 6.121 or > 6.121), and LGLRE (< 0.546 or > 0.546) from transverse T2WI. The logistic regression analysis demonstrated that higher DISS from sagittal fat-suppression T2WI and higher DISS and ENTR from transverse T2WI were independent predictors of local invasion. The logistic regression model incorporating the three independent predictors to differentiate T1/2 from T3/4 tumors achieved an AUC of 0.789 (95% CI, 0.703–0.859), with sensitivity of 88.10% and specificity of 61.29%. The corresponding ROC curve is shown in Figure 5.

**Table 5:** Results of multivariate logistic regression analysis.

**Method**	**Features**	**OR^a^**	**95% CI**	***P*-value**	**r_**s**_^b^**
**Sagittal fat-suppression**	DISS	7.937	1.411–47.381	0.036	0.351
**T2WI**					
	ENTR	3.535	0.959–24.122	0.197	0.338
	LGLRE	2.157	0.434–10.335	0.582	0.331
	Harr-V	1.337	0.324–3.296	0.990	−0.231
**Transverse T2WI**	DISS	8.261	1.639–54.293	0.028	0.354
	ENTR	9.884	1.911–61.474	0.016	0.370
	GLN	3.230	0.891–23.142	0.255	0.339
	LGLRE	1.990	0.417–8.526	0.911	0.302

a *OR, odds ratio*.

b *r_s_, Spearman rank correlation coefficient*.

## Discussion

The prognosis of patients with rectal cancer depends on many factors, including the depth that tumor lesion extends beyond or into muscularis propria, the involved lymph node number, invasion of the circumferential resection margin, tumor differentiation grade, and peritumor lymphangiovascular or neural invasion. This study investigated the correlation between the extent of local invasion in rectal cancer and texture features using preoperative sagittal fat-suppression and transverse T2WI data. The results demonstrated that texture features extracted from T2WI are potentially valuable imaging biomarkers in predicting the pathological T-stage of rectal cancer.

Extramural invasion is a key determinant for treatment decisions and an indication for NACT in patients with rectal cancer (19). Despite improvements in preoperative T staging through morphological analysis of high-resolution MRI, the accuracy of MRI remains unsatisfactory (20–22). More advanced and reliable techniques for detecting local invasion are necessary to determine the optimal treatment in patients with rectal cancer. TA can be used to quantitatively characterize the intratumoral heterogeneity by calculating the distribution of spatial arrangement of pixels (e.g., GLCM parameters) and gray-level values (histogram parameters) within a given ROI (23). The heterogeneity of a tumor arises from variations in extravascular extracellular matrix, angiogenesis, and cellularity as well as areas of hemorrhage and necrosis within the tumor (24). Increased tissue heterogeneity may lead to greater MRI heterogeneity in high-stage rectal tumors than in low-stage tumors. Yang et al. (16) reported that patients without regional lymph node metastasis had significantly higher energy, kurtosis, and skewness, and lower entropy on T2WI than those with lymph node metastasis. Jalil et al. (25) reported that texture features extracted from T2WI of rectal cancers were biomarkers of tumor response to NACT and long-term survival. Liu et al. (23) showed that high skewness and entropy values extracted from ADC maps were independent predictors of extramural invasion in rectal tumors. However, the correlation between texture features derived from sagittal fat-suppression combined with transverse T2WI and local invasion of rectal cancer remains unclear.

In this study, DISS, ENTR, GLN, and LGLRE extracted from both sagittal fat-suppression and transverse T2WI differed significantly between T1/2 and T3/4 tumors. These features showed significantly higher values in patients with stage T3/4 tumors than in those with T1/2 tumors. Furthermore, T3/4 tumors had a significantly lower Harr-V derived from sagittal fat-suppression T2WI than T1/2 tumors. Advanced rectal tumors are large, deeply infiltrated, and have high degrees of angiogenesis, necrosis, extracellular matrix, hemorrhage, and cellularity, which may lead to highly heterogeneous patterns on imaging modalities. Furthermore, rectal tumor invasion results in large areas of involvement, with additional necrotic or cystic areas or other abnormal tissues within lesions, which can lead to even more heterogeneity. Higher DISS values reflect greater local contrast. ENTR indicates the randomness of the intensity level distribution. A high GLN value represents differences in gray levels. High LGLRE reflects a high degree of disorder of the low gray-level distribution. Increases in these indicators represent the increased complexity of the texture in the lesion ROI, as well as increased tumor heterogeneity. These concepts are important for interpreting the present findings showing that high T-stage rectal tumors are more heterogeneous on T2WI than low T-stage tumors.

Multivariate analysis was applied to further investigate the correlation between the T-stage of rectal cancer and texture parameters. High DISS on sagittal fat-suppression T2WI and high DISS and ENTR on transverse T2WI were independent predictors of T-stage, with odds ratios of 7.937 for sagittal fat-suppression DISS, and 8.261 and 9.884 for DISS and ENTR on transverse T2WI, respectively. The AUC of the logistic regression model incorporating the three independent predictors was higher than that of significant texture features alone for differentiating rectal cancer T-stage.

The intraobserver variability for texture features extracted from both sagittal fat-suppression and transverse T2WI was evaluated. The results indicated excellent agreement between two radiologists with the respect to the measurement of texture features by a single-slice method, with ICCs ranging from 0.832 to 0.927. In fact, intraobserver variability was highly associated with the ROI delineation and slice selection, as texture feature calculation was conducted within the ROI from a single slice using in-house software with MATLAB 2018a. That means the approaches used for ROI definition are very important.

This study had several limitations. First, this was a retrospective study, which may lead to the selection bias. Second, the small sample size may limit the generalizability of the findings. Third, TA was applied to a single-slice MR image with the maximum tumor diameter rather than the whole tumor (26). Unlike most solid tumors, rectal tumors usually grow along the rectal wall and present an irregular shape; therefore, delineating the ROI using a single-slice method may not accurately represent the actual volume of the tumor. Fourth, DWI sequence was not used for TA. Probably, TA of DWI sequence could be more appropriate for the study aim since DWI reflects tumor biology; thus, TA of DWI images should be integrated in our further study. Fifth, the fact that all these patients came from the single center might limit the reproducibility of the results. The results should be further investigated by using data acquired using different scanners and imaging protocols. Hence, a future randomized multi-center prospective trial should be conducted. Finally, T3a- and T2-stage rectal cancers might have similar locoregional recurrence. T3-stage subgroups were not evaluated in this study and should be investigated in future studies.

## Conclusions

Texture features derived from preoperative T2WI were moderately associated with rectal cancer T-stage. In particular, high DISS on sagittal fat-suppression T2WI and high DISS and ENTR on transverse T2WI were independent predictors of high T-stage. These features could be helpful for assessing the T-stage of rectal cancer and thus for making treatment decisions.

## Data Availability Statement

All datasets presented in this study are included in the article/supplementary material.

## Ethics Statement

The studies involving human participants were reviewed and approved by Shengjing Hospital of China Medical University. The patients/participants provided their written informed consent to participate in this study.

## Author Contributions

JY: methodology, writing—original draft preparation, writing—review & editing, supervision, project administration, and funding acquisition. HL: validation. FW: investigation. All authors contributed to the article and approved the submitted version.

## Conflict of Interest

The authors declare that the research was conducted in the absence of any commercial or financial relationships that could be construed as a potential conflict of interest.

## References

[1] Valentini V van Stiphout RGPM Lammering G Gambacorta MA Barba MC Bebenek M. Nomograms for predicting local recurrence, distant metastases, and overall survival for patients with locally advanced rectal cancer on the basis of European randomized clinical trials. J Clin Oncol. (2011) 29:3163–72. 10.1200/JCO.2010.33.1595 21747092

[2] Glynne-Jones R Wyrwicz L Tiret E Brown G Rödel C Cervantes A Rectal cancer: ESMO clinical practice guidelines for diagnosis, treatment and follow-up. Ann Oncol. (2017) 28:iv22–40. 10.1093/annonc/mdx224 28881920

[3] Moreno CC Sullivan PS Mittal PK. MRI evaluation of rectal cancer: staging and restaging. Curr Probl Diagn Radiol. (2017) 46:234–41. 10.1067/j.cpradiol.2016.11.011 28089690

[4] Lu Z Wang L Xia K Jiang H Weng X Jiang J. Prediction of clinical pathologic prognostic factors for rectal adenocarcinoma: volumetric texture analysis based on apparent diffusion coefficient maps. J Med Syst. (2019) 43:331. 10.1007/s10916-019-1464-5 31701309

[5] Zhang XM Zhang HL Yu D Dai Y Bi D Prince MR. 3-T MRI of rectal carcinoma: preoperative diagnosis, staging, and planning of sphincter-sparing surgery. AJR Am J Roentgenol. (2008) 190:1271–8. 10.2214/AJR.07.2505 18430843

[6] Kim H Lim JS Choi JY Park J Chung YE Kim MJ. Rectal cancer: comparison of accuracy of local-regional staging with two- and three-dimensional preoperative 3-T MR imaging. Radiology. (2010) 254:485–92. 10.1148/radiol.09090587 20093520

[7] Heo SH Kim JW Shin SS Jeong YY Kang HK. Multimodal imaging evaluation in staging of rectal cancer. World J Gastroenterol. (2014) 20:4244–55. 10.3748/wjg.v20.i15.4244 24764662 PMC3989960

[8] Chitalia RD Kontos D. Role of texture analysis in breast MRI as a cancer biomarker: a review. J Magn Reson Imaging. (2019) 49:927–38. 10.1002/jmri.26556 30390383 PMC7077754

[9] Yoon HJ Kim Y Kim BS. Intratumoral metabolic heterogeneity predicts invasive components in breast ductal carcinoma in situ. Eur Radiol. (2015) 25:3648–58. 10.1007/s00330-015-3761-9 26063655

[10] Xia K Yin H Qian P Jiang Y Wang S Liver semantic segmentation algorithm based on improved deep adversarial networks in combination of weighted loss function on abdominal CT images. IEEE Access. (2019) 7:96349–58. 10.1109/ACCESS.2019.2929270

[11] Ytre-Hauge S Dybvik JA Lundervold A Salvesen ØO Krakstad C Fasmer KE. Preoperative tumor texture analysis on MRI predicts high-risk disease and reduced survival in endometrial cancer. J Magn Reson Imaging. (2018) 48:1637–47. 10.1002/jmri.26184 30102441

[12] Ueno Y Forghani B Forghani R Dohan A Zeng XZ Chamming's F. Endometrial carcinoma: MR imaging-based texture model for preoperative risk stratification-a preliminary analysis. Radiology. (2017) 284:748–57. 10.1148/radiol.2017161950 28493790

[13] Ahmed A Gibbs P Pickles M Turnbull L. Texture analysis in assessment and prediction of chemotherapy response in breast cancer. J Magn Reson Imaging. (2013) 38:89–101. 10.1002/jmri.23971 23238914

[14] Aker M Ganeshan B Afaq A Wan S Groves AM Arulampalam T. Magnetic resonance texture analysis in identifying complete pathological response to neoadjuvant treatment in locally advanced rectal cancer. Dis Colon Rectum. (2019) 62:163–70. 10.1097/DCR.0000000000001224 30451764

[15] Meng X Xia W Xie P Zhang R Li W Wang M. Preoperative radiomic signature based on multiparametric magnetic resonance imaging for noninvasive evaluation of biological characteristics in rectal cancer. Eur Radiol. (2019) 29:3200–09. 10.1007/s00330-018-5763-x 30413959

[16] Yang L Liu D Fang X Wang Z Xing Y Ma L. Rectal cancer: can T2WI histogram of the primary tumor help predict the existence of lymph node metastasis? Eur Radiol. (2019) 29:6469–76. 10.1007/s00330-019-06328-z 31278581

[17] Liu H Zhang C Wang L Luo R Li J Zheng H. MRI radiomics analysis for predicting preoperative synchronous distant metastasis in patients with rectal cancer. Eur Radiol. (2019) 29:4418–26. 10.1016/j.ejrad.2019.07.018 30413955

[18] DeLong ER DeLong DM Clarke-Pearson DL Comparing the areas under two or more correlated receiver operating characteristic curves: a nonparametric approach. Biometrics. (1988) 44:837–45. 10.2307/2531595 3203132

[19] van Gijn W Marijnen CA Nagtegaal ID Kranenbarg EM Putter H Wiggers T. Preoperative radiotherapy combined with total mesorectal excision for resectable rectal cancer: 12-year follow-up of the multicentre, randomised controlled TME trial. Lancet Oncol. (2011) 12:575–82. 10.1016/S1470-2045(11)70097-3 21596621

[20] Gollub MJ Lakhman Y McGinty K Weiser MR Sohn M Zheng J. Does gadolinium-based contrast material improve diagnostic accuracy of local invasion in rectal cancer MRI? A multireader study. AJR Am J Roentgenol. (2015) 204:160–7. 10.2214/AJR.14.12599 25615776 PMC4518447

[21] Peschaud F Cuenod CA Benoist S Julié C Beauchet A Siauve N. Accuracy of magnetic resonance imaging in rectal cancer depends on location of the tumor. Dis Colon Rectum. (2005) 48:1603–09. 10.1007/s10350-005-0051-7 15937629

[22] Heijnen LA Lambregts DM Mondal D Martens MH Riedl RG Beets GL. Diffusion-weighted MR imaging in primary rectal cancer staging demonstrates but does not characterise lymph nodes. Eur Radiol. (2013) 23:3354–60. 10.1007/s00330-013-2952-5 23821022

[23] Liu L Liu Y Xu L Li Z Lv H Dong N. Application of texture analysis based on apparent diffusion coefficient maps in discriminating different stages of rectal cancer. J Magn Reson Imaging. (2017) 45:1798–808. 10.1002/jmri.25460 27654307

[24] Davnall F Yip CS Ljungqvist G Selmi M Ng F Sanghera B. Assessment of tumor heterogeneity: an emerging imaging tool for clinical practice? Insights Imaging. (2012) 3:573–89. 10.1007/s13244-012-0196-6 23093486 PMC3505569

[25] Jalil O Afaq A Ganeshan B Patel UB Boone D Endozo R. Magnetic resonance based texture parameters as potential imaging biomarkers for predicting long-term survival in locally advanced rectal cancer treated by chemoradiotherapy. Colorectal Dis. (2017) 19:349–62. 10.1111/codi.13496 27538267

[26] Yang X Chen Y Wen Z Lu B Shen B Xiao X. Role of quantitative dynamic contrast-enhanced MRI in evaluating regional lymph nodes with a short-axis diameter of less than 5 mm in rectal cancer. AJR Am J Roentgenol. (2019) 212:77–83. 10.2214/AJR.18.19866 30354269

